# Proteomics of high-density lipoprotein subfractions and subclinical atherosclerosis in type 1 diabetes mellitus: a case–control study

**DOI:** 10.1186/s13098-023-01007-y

**Published:** 2023-03-11

**Authors:** Marcos Tadashi K. Toyoshima, Monique F. M. Santana, Amanda R. M. Silva, Gabriela B. Mello, Daniele P. Santos-Bezerra, Marisa F. S. Goes, Adriana A. Bosco, Bruno Caramelli, Graziella E. Ronsein, Maria Lucia Correa-Giannella, Marisa Passarelli

**Affiliations:** 1 Laboratorio de Lipides (LIM10), Hospital das Clinicas (HCFMUSP) Faculdade de Medicina, Universidade de Sao Paulo, Av. Dr. Arnaldo 455, Room 3305, Sao Paulo, SP 01246-000 Brazil; 2 Serviço de Onco-Endocrinologia, Instituto do Câncer do Estado de São Paulo Octávio Frias de Oliveira, Hospital das Clínicas da Faculdade de Medicina da Universidade de São Paulo, Sao Paulo, SP Brazil; 3 Departamento de Bioquímica, Instituto de Química, Universidade de São Paulo, São Paulo, Brazil; 4 Laboratório de Carboidratos e Radioimunoensaio (LIM18), Hospital das Clinicas (HCFMUSP) Faculdade de Medicina, Universidade de Sao Paulo, Sao Paulo, SP Brazil; 5 Laboratório de Aterosclerose, Instituto do Coração, Hospital das Clinicas (HCFMUSP) Faculdade de Medicina, Universidade de Sao Paulo, Sao Paulo, SP Brazil; 6 Unidade de Medicina Interdisciplinar em Cardiologia (UnMic), Instituto do Coração, Hospital das Clinicas (InCor, HCFMUSP) Faculdade de Medicina, Universidade de Sao Paulo, Sao Paulo, Brazil; 7 Programa de Pós-Graduação em Medicina, Universidade Nove de Julho, São Paulo, SP Brazil

**Keywords:** Type 1 diabetes mellitus, Cardiovascular disease, HDL, Proteomics, Vascular function

## Abstract

**Background:**

Subclinical atherosclerosis is frequently observed in type 1 diabetes (T1D) although the mechanisms and markers involved in the evolution to established cardiovascular disease are not well known. High-density lipoprotein cholesterol in T1D is normal or even high, and changes in its functionality and proteomics are considered. Our aim was to evaluate the proteomics of HDL subfractions in T1D and control subjects and its association with clinical variables, subclinical atherosclerosis markers and HDL functionality.

**Methods:**

A total of 50 individuals with T1D and 30 matched controls were included. Carotid-femoral pulse wave velocity (PWV), flow-mediated vasodilation (FMD), cardiovascular autonomic neuropathy (CAN), and ten-year cardiovascular risk (ASCVDR) were determined. Proteomics (parallel reaction monitoring) was determined in isolated HDL_2_ and HDL_3_ that were also utilized to measure cholesterol efflux from macrophages.

**Results:**

Among 45 quantified proteins, 13 in HDL_2_ and 33 in HDL_3_ were differentially expressed in T1D and control subjects. Six proteins related to lipid metabolism, one to inflammatory acute phase, one to complement system and one to antioxidant response were more abundant in HDL_2_, while 14 lipid metabolism, three acute-phase, three antioxidants and one transport in HDL_3_ of T1D subjects. Three proteins (lipid metabolism, transport, and unknown function) were more abundant in HDL_2_; and ten (lipid metabolism, transport, protease inhibition), more abundant in HDL_3_ of controls. Individuals with T1D had higher PWV and ten-year ASCVDR, and lower FMD, Cholesterol efflux from macrophages was similar between T1D and controls. Proteins in HDL_2_ and HDL_3_, especially related to lipid metabolism, correlated with PWV, CAN, cholesterol efflux, HDLc, hypertension, glycemic control, ten-year ASCVDR, and statins use.

**Conclusion:**

HDL proteomics can be predictive of subclinical atherosclerosis in type 1 diabetes. Proteins that are not involved in reverse cholesterol transport may be associated with the protective role of HDL.

**Supplementary Information:**

The online version contains supplementary material available at 10.1186/s13098-023-01007-y.

## Background

Type 1 diabetes (T1D) represents 5% to 10% of all diabetes cases [1] with an overall cardiovascular disease (CVD) risk—mainly manifested by coronary heart disease, cerebrovascular and peripheral artery disease—increased up to eight times in comparison to people without diabetes [2]. Good glycemic control in individuals with T1D is associated with a lower risk of microvascular complications and cardiovascular disease. Glycated hemoglobin (HbA1c) has been used as a standard measure for long-term (two to three months) glucose control, and serum fructosamine can be used to assess the glycemic control over the past two to three weeks [3]. The pathophysiology underlying CVD in T1D is not well understood, but its prevalence is related to increased HbA1c levels, diabetes duration, age, sex, and possibly to race and ethnicity. Besides, abnormal vascular function with increased artery calcification, endothelial dysfunction, and cardiovascular autonomic neuropathy (CAN) increase the susceptibility to macrovascular atherosclerotic disease [4].

Plasma lipids and lipoproteins are not classically altered in T1D as usually observed in type 2 diabetes (T2D) [5]. Nonetheless, changes in lipoprotein functionality are considered as putative contributors to CVD risk. HDL cholesterol (HDLc) plasma levels that are inversely related to CVD risk are normal or even higher in T1D [4, 6]. In this sense, alterations in HDL particle functionality ascribed to chemical modifications by glycation, oxidation, and autoantibodies and in its lipidomics, and proteomics have been described as pro-atherosclerotic by impairing its antiatherogenic properties [6, 7]. HDL are heterogeneous particles varying from 7 to 10 nm with different subpopulations. Pre-beta nascent HDL and lipid poor apolipoprotein A-I (apoA-I) remove excess cholesterol from peripheral cells, including arterial macrophages, by interacting with ATP binding cassette transporter A-1 (ABCA-1) in the first step of the reverse cholesterol transport (RCT). After esterification by the lecithin cholesterol acyltransferase, larger HDL particles are formed also removing cell cholesterol via ATP binding cassette transporter G-1 (ABCG-1). Cholesterol is driven to the liver by the HDL subfraction 2 (HDL_2_) that interacts with the scavenger receptor class B type 1 (SR-B1) or by the uptake of apoB-containing lipoproteins after receiving esterified cholesterol from HDL by the cholesteryl ester transfer protein (CETP). Cholesterol can be eliminated in the bile as free cholesterol, or after conversion into bile acids, which allows its excretion in feces [6, 8]. Besides its role along the RCT, HDL has antioxidant, anti-inflammatory, antiplatelet aggregation, and vasodilation actions that contribute to cardiovascular protection [8–10]. HDL is a cargo lipoprotein for microRNAs, several active lipid species, and proteins that do not directly relate to atherosclerosis but can modulate HDL function and even be a marker of its functionality [9]. Proteomics evidenced around 251 proteins associated with HDL with functions related to lipid metabolism, activation of the complement system, modulation of proteases, immunogenicity, and others [11]. It is conceivable that alterations in HDL´s protein cargo may interfere with its antiatherogenic properties that cannot be seen by simply measuring HDLc or apoA-I [12]. Nonetheless, there are only a few studies dealing with the proteome of HDL in T1D, all of them dealing with total HDL fraction and mostly with subjects with DM categorized according to glycemic control [13–15]. In the present investigation, the proteome of HDL subfractions (HDL_2_ and HDL_3_) was quantified by mass spectrometry using the parallel reaction monitoring (PRM) quantitative methodology. Subsequently, the proteome was related to HDL capacity in removing cell cholesterol, indicators of subclinical atherosclerosis—pulse wave velocity (PWV) and flow-mediated vasodilation (FMD) and ten-year atherosclerotic cardiovascular disease risk (ASCVDR) estimation in subjects with T1D in comparison to non-diabetes individuals.

This study aims to evaluate the proteomics of HDL subfractions in T1D subjects and controls and its association with clinical variables, subclinical atherosclerosis markers, and HDL functionality.

## Material and methods

This was a case–control study that enrolled 50 T1D and 30 non-diabetes control individuals matched by age, gender, and body mass index (BMI). The convenience sampling method was used in the study for the recruitment of individuals with T1D. Snowball sampling was used to recruit controls. The inclusion criteria were based on age ≥ 18 years, T1D diagnosis ≥ 5 years. Individuals with a personal history of clinically evident atherosclerotic macrovascular disease (coronary disease, peripheral arterial disease, or cerebrovascular disease), active smoking, or those who stopped smoking more than ten years ago, and triglycerides (TG) concentration > 400 mg/dL were not included. Participants signed an informed written consent form previously approved by The Ethical Committee for Human Research Protocols of the Hospital das Clinicas da Faculdade de Medicina da Universidade de São Paulo (#3.796.622; 01/09/2020), in accordance with the Declaration of Helsinki. The reporting of this study conforms to STROBE guidelines [16].

Peripheral blood was drawn after overnight fasting and plasma was immediately separated in a refrigerated centrifuge (4 °C). Plasma lipids [TG, total cholesterol, (TC), and HDLc], and plasma glucose were determined by enzymatic techniques (Labtest do Brasil, Minas Gerais, Brazil). HDLc was determined after precipitation of apoB-containing lipoproteins with 0.2% dextran sulfate/3 M magnesium chloride (v/v). Low-density lipoprotein (LDL) cholesterol (LDLc) was calculated by the Friedewald formula [17] and the estimated glomerular filtration rate (eGFR), using the CKD-EPI equation [18]. Fructosamine was determined by an automated colorimetric enzymatic method (Labtest do Brasil, Minas Gerais, Brazil). HbA1c was determined by high-performance liquid chromatography, certified by the National Glyco Hemoglobin Standardization Program (NGSP-USA).

### Vascular function tests

A subgroup of 30 subjects with T1D and 30 controls, matched by age, gender, and body mass index (BMI), underwent PWV and FMD tests. The PWV was determined in the carotid-femoral segment using a validated device (Complior®; Gonesse, France) [19, 20]. FMD tests were performed by a single researcher in accordance with the guidelines of the International Brachial Artery Reactivity Task Force (version 2002) [21]. Endothelium-dependent FMD and vascular smooth muscle response to the vasodilator isosorbide dinitrate (independent of the endothelium) were sequentially evaluated in the brachial artery. The brachial artery was accessed above the elbow crease and its diameter was verified by two independent observers, with the inter-observer correlation equal to 0.90 (*p* < 0.001). An ultrasound device (Sequoia Echocardiography System, version 6.0, Acuson Siemens^®^, Malvern, USA) equipped with a multifrequency linear transducer (7-12 MHz) and coupled to a computer specifically programmed to record and analyze this type of data was used. Reactive hyperemia (RH) was induced by inflating the sphygmomanometric cuff in a suprasystolic pressure (50 mmHg above systolic pressure), leading to transient ischemia due to occlusion of the brachial artery for two minutes, and subsequent cuff deflation. Data were obtained under baseline conditions, after induction of RH, and 5 min after oral administration of isosorbide dinitrate 10 mg. FMD(%) after RH was expressed as: [(RH Diameter − Basal Diameter)/Basal Diameter × 100]. Endothelium-independent vasodilation (EID;%) was expressed as: [(Post-nitrate Diameter − Pre-nitrate Diameter)/Pre-nitrate Diameter]. Measurement of basal and after RH indution brachial artery diameter were performed in the T1D individuals and controls, but FMD after nitrate was not performed in controls, considering the risk of hypotension in individuals with supposedly full production of nitric oxide. Subclinical atherosclerosis was defined as the stage that precedes clinical atherosclerotic vascular disease. As the results of the PWV and FMD vascular tests were considered continuous variables, the results were evaluated by comparing the T1D group and the controls regarding the difference in vascular parameters.

### Cardiovascular autonomic tests

Sympathetic and parasympathetic cardiac function assessment was performed only in individuals with T1D, by analyzing seven tests that include Ewing's standardized tests and spectral analysis performed by the Poly-Spectrum software (version 4.8.143; Neurosoft^®^, Ivanovo, Russia). Three or more altered tests indicated the presence of CAN [22, 23].

### Cardiovascular risk estimate

The validated tools T1 Risk Engine [24] and QRISK3 [25] (available on the internet) were used to estimate ten-year ASCVDR in T1D.

### Lipoproteins isolation

HDL subfractions [HDL_2_; density (d) = 1.063–1.125 g/mL, and HDL_3_; d = 1.125–1.21 g/mL] were isolated by discontinuous density gradient ultracentrifugation (100.000* g*, 4 °C, 24 h, SW40 rotor; Beckman ultracentrifuge) [26]. Samples were dialyzed against phosphate-buffered saline containing EDTA (PBS) and kept frozen at − 80 °C. LDL (d = 1.019–1.063 g/mL) was isolated from a pool of healthy plasma donors and acetylated as previously described by Basu et al. [26], followed by extensive dialysis.

### HDL proteolytic digestion

Ten micrograms of HDL protein were digested with trypsin according to the study of Silva et al. [27]. Robustness of the PRM methodology was controlled by using angiotensin peptide (DRVYIHPFHL, 0.2 pmol/µL) spiked in each sample as global internal standard. A coefficient of variance of 13% was attained.

### Targeted proteomic analyses

Fifty nanograms of digested HDL proteins were quantified by parallel reaction monitoring (PRM), as described by Silva et al. [27]. An inclusion list containing m/z of precursor peptides of interest (3-min window) and corresponding retention times was created by the Skyline software [28]. MS proteomics data have been deposited to the Mass Spectrometry Interactive Virtual Environment (MassIVE) [29].

Sixty-seven proteins were identified, but reduced to 45 after eliminating proteins that could be potential contaminants or in low abundance. Peptides susceptible to ex vivo modification (e.g., methionine-containing peptides) were also avoided, and only peptides satisfactorily detected (with a good chromatographic peak, containing at least four coeluted transitions, and with mass error < 10 ppm) were included in the final analysis.

### Determination of ^14^C-cholesterol efflux from macrophages

Procedures with mice were approved by the Institutional Animal Care and Research Advisory Committee of Faculdade de Medicina da Universidade de Sao Paulo (COBEA-CEUA FMUSP 071/17) and were performed following the U.S. National Institutes of Health Guide for the Care and Use of Laboratory Animals. Mice were euthanized with an intraperitoneal overdose of ketamine hydrochloride (300 mg/kg of body weight) and xylazine hydrochloride (30 mg/kg of body weight). Six-week-old male C57BL/6J mice were utilized for the isolation of bone marrow-derived cells as previously described [30]. Bone marrow-derived macrophages were overloaded with acetylated LDL (50 µg/mL DMEM) and ^14^C-cholesterol (0.3 µCi/mL), for 48 h. HDL subfractions (50 µg/mL) were utilized as cholesterol acceptors in 6-h incubations. The percentage of cholesterol efflux was calculated as: [^14^C-cholesterol in the medium/(^14^C-cholesterol in the medium + ^14^C-cholesterol in cells) × 100]. Control incubations were performed in the absence of HDL, and results subtracted from those obtained in the presence of HDL, as previously described [31].

### Statistical analysis

Descriptive analyses were expressed as mean ± SD or median (25th-75th percentiles), and qualitative variables expressed as absolute (n) and relative (%) frequencies. Pearson's chi-square test was used for categorical variables, and Student's t-test or Mann–Whitney for continuous variables. Data normality was accessed by the Shapiro–Wilk and Kolmogorov–Smirnov tests. Proteins in HDL were differentiated by the Wilcoxon independent and paired nonparametric test. Peptide abundances were log10 transformed and *p* values corrected by the Benjamini–Hochberg method. A corrected *p*-value threshold was calculated, and only proteins above that were considered significantly different. The odds ratio (95% confidence interval) was calculated for the association of proteins and discriminant analysis was performed to determine the discriminatory capacity of proteomics data for the both groups. Correlations for linear continuous or monotonous relationships between continuous or ordinal variables were determined, respectively, by Pearson's or Spearman's correlation. No imputation method was used to address missing data. R Studio version 1.1.463 (RStudio. Inc.), and SPSS version 21.0 (SPSS Inc., IBM) were used for analysis. A *p* < 0.05 was considered significant.

## Results

Clinical data from 50 T1D and 30 controls are presented in Table 1. Groups were similar regarding age, sex, BMI, abdominal circumference, HDLc, and eGFR. HbA1c and fructosamine levels were higher in T1D, and plasma TC, LDLc, and TG in controls. Retinopathy (48%) and CAN (30%) were the most prevalent complications in T1D. Forty percent of T1D used statins, and 15% had concomitant hypertension. Subjects with T1D and hypertension had longer duration of diabetes, and presence of albuminuria, retinopathy, and statins use. Glycemic control (HbA1c < 8.5% and ≥ 8.5%) was only statistically associated with albuminuria.

**Table 1:** Clinical, antropometric data and vascular function from subjects with type 1 diabetes and controls

	T1D n = 50	Controls n = 30	*p*
Female sex (%)	50	50	–
Age (years)	34 (29–40)	34 (30–46)	0.474
BMI (kg/m^2^)	22.9 (20.3–26.6)	24.4 (23.2–26.60)	0.077
Abdominal circumference (cm)	89 (80–96)	86 (79–95)	0.530
Time of T1D (years)	22 (16 – 30)	NA	
Age at T1D diagnosis (years)	11.5 (6 – 18)	NA	
Hypertension, n (%)	15 (30%)	0	
HbA1c (%)	8.3 (6.9–9.3)	5.4 (5.0–5.5)	< 0.001
Fructosamine (µmol/L)	362 (336–421)	226 (215–237)	< 0.001
TC (mg/dL)	163 (145–184)	186.5 (153–199)	0.017
HDLc (mg/dL)	57 (48–67)	56.5 (46–68)	0.709
LDLc (mg/dL)	86.5 (75–104)	97 (86–120)	0.021
TG (mg/dL)	69 (57–93)	86 (68–134)	0.036
eGFR (mL/min/1.73m^2^)	103.5 (90.2–117.2)	101.0 (89.7–100.1)	0.538
Albuminuria (mg/g creatinine)	6.1 (4.2–16.4)	NA	
Retinopathy. n (%)	24 (48%)		
Albuminuria. n (%)			
A1: < 30 mg/g creatinine	40 (80%)		
A2: 30–300 mg/g creatinine	8 (16%)		
A3: > 300 mg/g creatinine	2 (4%)		
Stages of kidney function—eGFR (mL/min/1.73m^2^)			
G1: ≥ 90	38 (76%)		
G2: 60–89	8 (16%)		
G3a: 45–59	2 (4%)		
G3b: 30–44	1 (2%)		
G4: 15–29	0		
G5: < 15	1 (2%)		
Peripheral neuropathy, n (%)	6 (12%)		
CAN, n (%)	15 (30%)		
Medications, n (%)			
Statins	20 (40%)		
Atorvastatin	10 (20%)		
Simvastatin	10 (20%)		
ACEi	12 (24%)		
ARB	7 (14%)		
Thiazide Diuretics	7 (14%)		
Spironolactone	1 (2%)		
Beta blocker	2 (4%)		
CCB	1 (2%)		
Hydralazine	1 (2%)		
Ten-year ASCVDR estimate (%)			
Steno T1 Engine	6.9 (4.2–15.6)		
QRISK3	4.5 (2.0–7.6)		

	n = 30	n = 30	
PWV (m/s)	7.7 (6.9—8.4)	6.7 (6.1–7.3)	0.008
Brachial artery diameter (mm)	3.8 (3.2–4.3)	3.7 (3.3–4.5)	0.781
FMD after RH (%)	2.7 (− 0.1 to 8.6)	7.4 (2.7–9.7)	0.047
FMD after nitrate (%)	18.4 (14.1–4.6)	–	

Categorical variables: Absolute (n) and relative (%) frequency, Pearson’s Chi-square test. Continuous variables: Median (25th-75th percentile), Mann Whitney Test

*ACEi* angiotensin-converting enzyme inhibitors, *ARB* angiotensin receptor blocker, *ASCVDR* atherosclerotic cardiovascular disease risk, *BMI* body mass index, *CAN* cardiovascular autonomic neuropathy, *CCB* calcium channel blockers, *eGFR* estimated glomerular filtration rate, *FMD* flow-mediated vasodilation, *HDLc* HDL cholesterol, *LDLc* LDL cholesterol, *NA* not available, *PWV* pulse wave velocity, *RH* reactive hyperemia, *T1D*: type 1 diabetes, *TG* triglycerides, *TC* total cholesterol

**Fig. 1:**
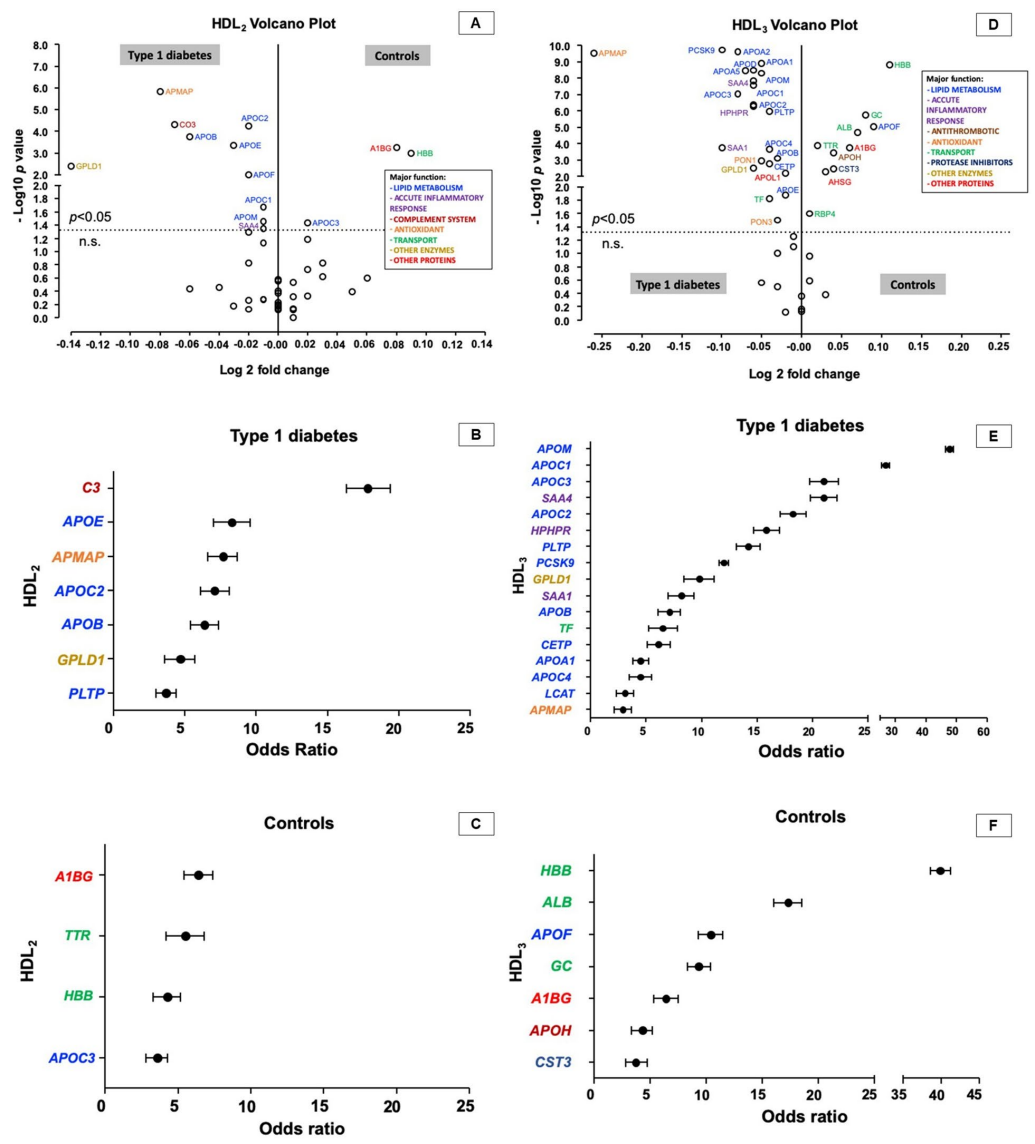
Differences in HDL_2_ and HDL_3_ protein abundance between T1D and controls. Volcano Plot indicating differences of protein abundance (log2 fold change) detected in HDL_2_ (**A**) and HDL_3_ (**D**) (significance in −log_10_) and odds ratio with 95% confidence interval for the association of HDL_2_ and HDL_3_ proteomics in T1D (**B** for HDL_2_ and **E** for HDL_3_) and controls (**C** for HDL_2_ and **F** for HDL_3_). Proteins are indicated by gene name abbreviation. *AHSG* alpha-2-Heremans-Schmid glycoprotein or fetuin-A, *ALB* albumin, *APMAP* adipocyte plasma membrane-associated protein, *apoH* apolipoprotein H or beta 2 glycoprotein 1, *A1BG* glycoprotein alpha-1B, *CETP* cholesteryl ester transfer protein, *CST3* cystatin C, *GC* vitamin D binding protein or group-specific component, *GPLD1* phosphatidylinositol-glycan-specific phospholipase D, *HBB* hemoglobin subunit beta, *HPHPR* haptoglobin or haptoglobin related protein, *LCAT* lecithin cholesterol acyltransferase, *n.s.* not significant, *PCSK9* proprotein convertase subtilisin/kexin type 9, *PLTP* phospholipid transfer protein, *PON1* paraoxonase-1, *PON3* paraoxonase-3, *RBP4* retinol binding protein type 4, *SAA1* serum amyloid A1, *SAA4* serum amyloid A4, *TF* Transferrin, *TTR* transthyretin

Forty-five proteins were selected and quantified in association with HDL_2_ and HDL_3_ of T1D and controls. From those, 18 were primarily associated with lipid metabolism [apo(a), apoA-I, apoA-II, apoA-IV, apoA-V, apoB, apoC-I, apoC-II, apoC-III, apoC-IV, apoD, apoE, apoF, apoM, CETP, LCAT, PCSK9, and PLTP], five related to acute inflammatory response (SAA1, SAA4, HP, HPHPR, and Orm1), one with complement system (C3), one antithrombotic (apoH), four antioxidants (APMAP, clusterin, PON1, and PON3), six transport proteins (ALB, GC, IGFALS, RBP4, TF, and transthyretin), five protease inhibitors (AHSG, AMBP, CST3, A1AT, and vitronectin), two enzymes not related with lipid metabolism (GPLD1 and PCYOX) and three with not well-known function (apoL-I, A1BG, and HBB) (Table 2). The differences between the abundance of proteins detected in HDL_2_ between T1D and controls are shown in the Volcano Plot (Fig. 1A). Ten proteins (APMAP, apoB, apoC-I, apoC-II, apoE, apoF, apoM, C3, GPLD1 and SAA4) were more abundant in HDL_2_ from T1D, and three (A1BG, apoC-III and HBB) in controls. The T1D was more associated with greater abundance of seven proteins (C3, apoE, APMAP, apoC-II, apoB, GPLD-1, and PLTP) (Fig. 1B). In the controls, the association was with four proteins (A1BG, transthyretin, HBB, and apoC-III) (Fig. 1C). Six proteins (APMAP, apoC-II, apoC-III, C3, HBB, and PLTP) were found to discriminate the HDL_2_ proteomics between T1D and controls. Thirty-three proteins were differentially expressed in HDL_3_ from T1D and controls (Fig. 1D), being 23 (APMAP, apoA-I, apoA-II, apoA-V, apoB, apoC-I, apoC-II, apoC-III, apoC-IV, apoD, apoE, apoL-I, apoM, CETP, GPLD1, HPHPR, PCSK9, PLTP, PON1, PON3, SAA1, SAA4, and TF) more abundant in T1D, and ten (A1BG, AHSG, ALB, apoF, apoH, CST3, GC, HBB, RBP4, and transthyretin) in controls. T1D was more associated with the abundance of 17 proteins (apoM, apoC-I, apoC-III, SAA4, apoC-II, HPHPR, PLTP, PCSK9, GPLD1, SAA4, apoB, TF, CETP, apoA-I, apoC-IV, LCAT, and APMAP) (Fig. 1E), and the controls with seven proteins (HBB, ALB, apoF, GC, A1BG, apoH, and CST3) (Fig. 1F). Nonetheless, only apoA-I, HBB, APMAP, LCAT, and PCSK9 had discriminatory capacity in HDL_3_ proteomics between groups.

**Table 2:** Proteins evaluated in HDL in type 1 diabetes and controls, divided by their main functions

Abbreviation	Protein name	Gene
Lipid metabolism		
Apo(a)	Apolipoprotein (a)	*LPA*
ApoA-I	Apolipoprotein A-I	*APOA1*
ApoA-II	Apolipoprotein A-II	*APOA2*
ApoA-IV	Apolipoprotein A-IV	*APOA4*
ApoA-V	Apolipoprotein A-V	*APOA5*
ApoB	Apolipoprotein B	*APOB*
ApoC-I	Apolipoprotein C-I	*APOC1*
ApoC-II	Apolipoprotein C-II	*APOC2*
ApoC-III	Apolipoprotein C-III	*APOC3*
ApoC-IV	Apolipoprotein C-IV	*APOC4*
ApoD	Apolipoprotein D	*APOD*
ApoE	Apolipoprotein E	*APOE*
ApoF	Apolipoprotein F or lipid transfer inhibitor protein	*APOF*
ApoM	Apolipoprotein M	*APOM*
CETP	Cholesteryl ester transfer protein	*CETP*
LCAT	Lecithin-cholesterol acyltransferase	*LCAT*
PCSK9	Proprotein convertase subtilisin/kexin type 9	*PCSK9*
PLTP	Phospholipid transfer protein	*PLTP*
Accute inflammatory response		
SAA1	Serum amyloid A type 1	*SAA1*
SAA4	Serum amyloid A type 4	*SAA4*
HP	Haptoglobin	*HP*
HPHPR	Haptoglobin or haptoglobin related protein	*HP/ HPR*
Orm1	Alpha-1 glycoprotein 1 or orosomucoid	*ORM1*
VTN	Vitronectin	*VTN*
Complement system		
C3	Complement C3	*C3*
Antithrombosis		
ApoH	Apolipoprotein H or beta-2-glycoprotein 1	*APOH*
Antioxidant		
APMAP	Adipocyte plasma membrane-associated protein	*APMAP*
ApoJ/ CLU	Clusterin, apolipoprotein J or sulfated glycoprotein 2	*CLU*
Pon1	Paraoxonase-1	*PON1*
Pon3	Paraoxonase-3	*PON3*
Transport proteins		
Alb	Albumin	*ALB*
GC	Vitamin D binding protein or Group-specific component	*GC*
HBB	Hemoglobin subunit beta	*HBB*
IGFALS	Acid-labile subunit	*IGFALS*
RBP4	Retinol binding protein type 4	*RBP4*
TF	Serotransferrin	*TF*
TTR	Transthyretin	*TTR*
Protease inhibitors		
A1AT	Alpha-1 antitrypsin	*SERPINA1*
AHSG	Alpha-2-Heremans-Schmid glycoprotein or fetuin-A	*AHSG*
AMBP	Alpha-1 microglobulin bikunin precursor	*AMBP*
CST3	Cystatin C	*CST3*
VTN	Vitronectin	*VTN*
Enzymes (do not involved in lipid metabolism)		
GPLD1	Phosphatidylinositol-glycan-specific phospholipase D	*GPLD1*
PCYOX	Prenylcysteine oxidase 1	*PCYOX1*
Other proteins		
ApoL-I	Apolipoprotein L-I	*APOL1*
A1BG	Alpha-1-B glycoprotein	*A1BG*

Forty-five proteins were selected and quantified in HDL_2_ e HDL_3_ in T1D and controls and were divided by major functions

In HDL_2_ of T1D, the amount of apoB and Lp(a) was negatively correlated, while PLTP, PON1, and A1AT were positively correlated with plasma HDLc. In controls, apoF presented a negative correlation, and apoA-I, GPLD1, PLTP, and PON1 had a positive association with HDLc. Only two proteins presented a similar correlation in both groups (PLTP and PON1). For HDL_3_, the abundance of three proteins (C3, IGFALS, and PON1) was positively correlated with HDLc of T1D. In controls, three proteins in HDL_3_ (AMBP, apoF and apoH) were negatively correlated with HDLc; while five proteins (C3, clusterin, IGFALS, LCAT, and PON1) were positively correlated. Only two proteins presented a similar correlation in both groups (C3 and IGFALS). PON1 correlated well in controls, but not in T1D (data not shown).

APMAP, apoB, apoC-I, C3, and SAA4 in HDL_2_ was greater in T1D with HbA1c ≥ 8.5%, while IGFALS was less abundant in those subjects. In HDL_3_, 12 proteins (APMAP, apoA-I, apoA-II, apoA-V, apoC-I, apoC-II, apoC-III, apoD, apoM, HPHPR, PCSK9, and SAA4) were more abundant in individuals with HbA1c ≥ 8.5%, while the content of eight proteins (A1AT, A1BG, Alb, apoF, GC, HBB, RBP4, and transthyretin) was greater in T1D with HbA1c < 8.5% (Additional file 1: Table S1). Regarding the categorization by statin use, a higher abundance of C3, HBB and apo(a), and a lower abundance of apoM, IGFALS, and PON3 in HDL_3_ was observed (Additional file 1: Table S1).

The presence of CAN, assessed in T1D, was associated with a higher abundance of AMBP, ApoB, Lp(a), Orm1, and transthyretin in HDL_2_, and lower content of clusterin, HBB, and PON3 in HDL_3_ (data not shown). A positive correlation was observed between A1BG content in HDL_2_ with ten-year ASCVDR assessed by the QRISK3; AMBP was positively, and apoE in HDL_2_ negatively correlated with both QRISK3 and Steno T1 Engine (Additional file 1: Table S1). Also, the ten-year ASCVDR by Steno T1 Engine showed a positive correlation with AHSG, AMBP, and apoH in HDL_3_, and a negative correlation with apoC-I, apoE, apoL-I, and HPHPR. The ten-year ASCVDR calculated by QRISK3 presented a positive correlation with eight proteins in HDL_3_ (A1BG, AHSG, ALB, AMBP, apoA-IV, apoH, RBP4, and transthyretin) and negative with apoC-I, apoE, apoL-I, and apoM (data not shown).

Thirty individuals in T1D and 30 in controls underwent PWV and post-reactive hyperemia FMD tests. T1D presented higher PWV than controls. In the assessment of endothelial function, the basal diameter of the brachial artery was similar, but FMD after reactive hyperemia was lower in the T1D than controls (Table 1). In both groups, PWV, but not FMD, correlated with ten-year ASCVDR by Steno T1 Engine Risk and QRISK3. CAN and HDLc were not associated with changes in vascular function.

In T1D, six proteins in HDL_2_ (apoL-I, A1BG, apoA-II, apoB, apo(a),and SAA4) positively correlated with PWV while PCSK9 and clusterin presented an inverse association. Post-RH FMD was positively correlated with apoA-I and PCYOX. In the controls, HBB and Orm1 showed a negative correlation with PWV, and post-RH FMD was not related to any protein associated with HDL_2_. After nitrate, A1AT, apoA-IV, apoF, clusterin, PCSK9, and vitronectin negatively correlated with FMD, while IGFALS directly related to FMD in T1D. In the controls, a negative association was observed between HBB and Orm1 with PWV, with no association of any protein with FMD (Table 3).

**Table 3:** Correlation between vascular function and HDL_2_ and HDL_3_ proteomics in subjects with T1D and controls

	PWV		FMD after reactive hyperemia		FMD after nitrate	
	HDL_2_	HDL_3_	HDL_2_	HDL_3_	HDL_2_	HDL_3_
T1D						
A1AT		− 0.38 (0.04)			− 0.47 (0.01)	
A1BG	0.49 (< 0.01)					
ApoA-I		− 0.38 (0.04)	0.41 (0.02)			
ApoA-II	0.49 (< 0.01)					
ApoA-IV					− 0.40 (0.04)	
ApoB	0.41 (0.03)					
ApoE		− 0.40 (0.03)				
ApoF					− 0.44 (0.02)	
ApoL-I	− 0.60 (< 0.01)	− 0.44 (0.02)				
C3		− 0.48 (0.01)				
CLU	− 0.39 (0.04)	− 0.51 (< 0.01)			− 0.39 (0.04)	
HBB		− 0.38 (0.04)				
GPLD1				− 0.42 (0.02)		
IGFALS		− 0.47 (0.01)			0.43 (0.03)	
LCAT		− 0.49 (< 0.01)				
Lp(a)	0.46 (0.01)					
PCSK9	0.51 (< 0.01)				− 0.43 (0.03)	
PCYOX			0.36 (0.05)			
PON1		− 0.47 (0.01)				
SAA4	0.43 (0.02)					
VTN					− 0.39 (0.04)	
Controls						
ApoC- IV		− 0.36 (0.05)				
CETP		0.38 (0.04)		− 0.37 (0.05)		
HBB	− 0.38 (0.04)					
Orm1	− 0.39 (0.03)					
RBP4		0.38 (0.04)				

Spearman’s correlation, expressed as a correlation coefficient (r) and the significance of the correlation is expressed as a *p* value, in parentheses. *A1AT* alpha-1 antitrypsin, *A1BG* A1BG alpha-1-B glycoprotein, *Apo* apolipoprotein, *C3* complement C3, *CETP* cholesteryl ester transfer protein, *CLU* clusterin or apolipoprotein J, *FMD* flow-mediated vasodilation, *GPLD1* phosphatidylinositol-glycan-specific phospholipase D, *HBB* hemoglobin subunit beta, *IGFALS* acid-labile subunit, *LCAT* lecithin cholesterol acyltransferase, *Lp(a)* apolipoprotein(a), *Orm1* alpha-1 glycoprotein 1 or orosomucoid, *PCSK9* proprotein convertase subtilisin/kexin type 9, *PCYOX* prenylcysteine oxidase 1, *PON1* paraoxonase-1, *PWV* pulse wave velocity, *RBP4* retinol binding protein type 4, *SAA4* serum amyloid A type 4, *T1D* type 1 diabetes, *VTN* vitronectin

In HDL_3_ of T1D, nine proteins (clusterin, A1AT, apoA-I, apoE, apoL-I, complement C3, HBB, IGFALS, LCAT, and PON1) were inversely related to PWV. FMD after RH and nitrate were inversely correlated with GPLD1. In the controls, apoC-IV was negative, while CETP and RBP4 positively correlated with PWV. Regarding post-RH FMD, a negative association was observed with CETP (Table 3). CAN was associated with the higher abundance of AMBP, apoB, Lp(a), Orm, and transthyretin in HDL_2_, and lower content of clusterin, HBB, and PON3 in HDL_3_. In HDL_2_, A1BG presented a positive correlation with QRISK3; while and AMBP and apoE, respectively, had a positive and a negative correlation with QRISK3 and Steno 1 Engine. Regarding HDL_3_, AHSG, AMBP, and apoH positively correlated, while apoC-I, apoE, apoL-I e HPHPR, negatively correlated with Steno T1 Engine. For QRISK3, the positive association was observed with A1BG, AHSG, ALB, AMBP, apoA-IV, apoH, RBP4, and transthyretin) and a negative correlation with apoC-I, apoE, apoL-I, and apoM.

The percentage of 6-h cholesterol efflux from macrophages was similar between T1D [HDL_2_: 24.1% (18.0–30.2); HDL_3_: 18.8% (14.8–24.4)] and controls [HDL_2_: 22.1% (18.5–25.7); HDL_3_: 18.5% (16.1–22.6)]. It was not observed association between clinical variables with cholesterol efflux capacity, except for an inverse correlation between the percentage of HDL_2_-mediated efflux with albuminuria in T1D (r = − 0.339; *p* = 0.02). Three proteins in HDL_3_ of T1D negatively correlated with cholesterol efflux: A1AT (r = − 0.33; *p* = 0.03), GC (r = − 0.32; *p* = 0.03), and TF (r = − 0.31; *p* = 0.04). In HDL_3_ of controls, eight were negatively correlated with cell cholesterol efflux: A1BG (r = − 0.41; *p* = 0.03), AHSG (r = − 0.48; *p* = 0.01), apoF (r = − 0.46; *p* = 0.01), apoH (r = − 0.46; *p* = 0.01), CST3 (r = − 0.38; *p* = 0.04), GC (r = − 0.46; *p* = 0.01), RBP4 (r = − 0.434; *p* = 0.02), and transthyretin (r = − 0.60; *p* = 0.01). Only RBP4 in HDL_2_ of controls positively correlated with cell cholesterol removal (r = 0.46; *p* = 0.02).

## Discussion

Subclinical atherosclerosis is more frequently observed in T1D subjects including increased intima-media thickness, artery calcification, and abnormal vascular function, namely PWV and FMD [32–35]. Nonetheless, the mechanisms involved in the evolution to clinical established CVD and possible markers are not well known. HDL protein signature may provide new insights on the modulation of HDL functionality that may not be evident by the classical metrics of this lipoprotein in plasma. In the present investigation, it was demonstrated a remodeling in HDL proteomics in T1D as compared to controls which related to markers of subclinical atherosclerosis.

T1D presented similar concentrations of plasma HDLc and lower levels of TG, LDLc, and TC as compared to controls. On the other hand, subclinical atherosclerosis markers, such as greater PWV and reduced FMD, were greater in T1D. Most studies that evaluated arterial stiffness in DM were conducted in T2D and those with T1D were carried out mainly in children, with different results and assessment methods [14, 34, 36]. Increased arterial stiffness observed in T1D corroborates other studies carried out with individuals of similar ages that also showed an association of higher PWV with inflammatory biomarkers in T1D [37]. The concomitance of other cardiovascular risk factors, such as hypertension and dyslipidemia, was associated with greater arterial stiffness (PWV), but not with endothelial dysfunction (FMD). Interestingly, glycemic control was not associated with abnormal vascular function, although it is noted to mention that the current investigation is a cross-sectional study, and HbA1c levels represent the mean glycemic rate for the last three months. Both the increased values of HbA1c and serum fructosamine in individuals with T1D suggest that there were no acute changes in glycemic control. CAN that may affect up to 30% T1D individuals [38, 39] was not associated with vascular test results, although Liatis et al. [40] demonstrated that cardiovascular autonomic function, especially parasympathetic activity, is related to arterial stiffness in individuals with T1D. The two cardiovascular risk assessment tools validated in T1D—Steno T1 Engine and QRISK3—indicated an association between increased PWV and reduced FMD with higher ten- year ASCVDR [37].

HDLc were not associated with vascular dysfunction in T1D reinforcing the hypothesis that HDLc may not represent the best prediction for CVD in DM. The proteome analysis revealed differences in abundance of 13 peptides in HDL_2_, and 33 in HDL_3_ between the T1D and controls. A study demonstrated a distinct profile of proteins carried by total HDL fraction in T1D as compared to controls, including apoA-IV, apoE, and apoD, fibrinogen, albumin, and others, together with irreversible post-translational modifications such as oxidation, deamidation, and glycation. Those changes were related to a reduced ability of serum from subjects with T1D in removing cholesterol from macrophages as well as a diminished antioxidant capacity of HDL from subjects with T1D, independently of the glycemic control and HDLc [13]. In another case–control study, a labeled-free SWATH peptide quantification revealed 78 proteins bound to HDL that were differentially expressed in youth people with T1D in comparison to controls although no association with HDL functionality (^3^H-cholesterol efflux analysis) was made [14]. In a subgroup of subjects enrolled in Diabetes Control and Complications Trial/Epidemiology of Diabetes Intervention and Complications Study (DCCT/EDIC), 46 proteins were quantified in HDL by isotope dilution tandem-mass spectrometry, and after stringent analysis, eight proteins were associated with albuminuria. Particularly, PON1 in HDL was inversely correlated with coronary calcium score, and positively with albumin excretion rate [41]. Nine proteins in HDL_2_, and 22 in HDL_3_ whose difference in abundance were observed in the present investigation were not reported in the two case–control studies described above. The AMBP, albumin, and apoA-II were in agreement with at least one other study. ApoA-IV, apoH, A1BG, and C3 were also found in at least one of the two studies and had a significant difference between the groups, but the result was not in agreement regarding the group with greater abundance [14, 15].

Many proteins directly involved in lipid metabolism presented a greater content in HDL subfractions in T1D and may represent interplay of metabolic pathways that modulate lipid metabolism in T1D. Noteworthy that the abundance of PLTP, apoA-I, LCAT and PCSK9 had a discriminatory power between T1D and controls. PLTP is responsible for HDL remodeling and elevated PLTP activity is reported in T1D being considered as a contributor for enhancing apoA-I in fused HDL particles. ApoA-I mediates many atheroprotective actions of HDL, including RCT, antioxidative and anti-inflammatory activities, although increased modification of apoA-I by glycation, oxidation and others impairs its functionality contributing to HDL loss of function in diabetes [42]. The LCAT activity in T1D is reported as increased according to hyperglycemia [43] or unchanged [44] and its role in atherogenesis is controversial. Shao et al. [41] demonstrated that LCAT was inversely related to coronary calcium score in T1D individuals which agrees with the negative correlation observed between this enzyme with PWV observed in the present investigation. Interestingly, PCSK9 was more abundant in HDL_3_ of T1D especially in those with HbA1c values ≥ 8.5% and its amount in HDL_2_ was associated with endothelial dysfunction. Plasma levels of PCSK9 were found increased in T1D subjects, which correlated with glycemic control [45]. Moreover, PCSK9 was associated with smaller HDL particles in T1D individuals with a poor glycemic control, although its role in HDL is not well established [46]. Other proteins related to lipid metabolism abundant in HDL subfractions of T1D included apoA-II, apoC-III, apoA-V, and apoD apoC-I, and apoC-II, that correlated with glycemic control, and apoA-IV, inversely correlated with PWV. Interestingly, apoC-III was greatly expressed in HDL_2_ of controls and HDL_3_ of T1D subjects. This apolipoprotein reflects insulin sensitivity and negatively modulates LPL activity driving plasma levels of triglycerides and being a target of drugs for hypertriglyceridemia control. Moreover, it drives VLDL secretion and relates to inflammatory processes in the vasculature and in the pancreas. In the present investigation, the differential expression of apoC-III in HDL was not correlated to HDL functionality (^14^C-cholesterol efflux analysis) or vascular tests, which deserves further exploration.

Acute inflammatory proteins such as SAA1, SAA4 that presented a discriminatory power between both groups were related by others to the impairment in RCT and anti-inflammatory activity of HDL [6, 47, 48]. Complement C3 in HDL_3_ that in the present study was inversely related to PWV, was described as enhanced in the HDL proteome of subjects with established CVD [49] and elevated in the HDL proteome in T1D (11). PON1 and PON3 were higher in HDL_3_ being PON1 inversely related to PWV. In the DCCT/ EDIC those enzymes were inversely related to calcium coronary score [43].

Curiously, the hemoglobin subunit beta (HBB) in both HDL_2_ and HDL_3_ had a discriminatory capacity for T1D and controls, being HBB reduced in T1D and with a negative correlation with PWV. APMAP was also discriminatory but more abundant in T1D with worse glycemic control. Finally, the clusterin content in HDL_2_ and HDL_3_ was positive and negative correlated with, respectively, PWV and FMD. Moreover, clusterin was negatively correlated with CAN, agreeing with its role in preventing familial amyloidotic polyneuropathy [50].

The PRM analysis is a very sensitive methodology making detectable the presence of very small amounts of proteins such as apoB and apo(a) in HDL, as the hydrated density of large HDL_2_ is similar to the densities of lipoprotein (a) and LDL [27].

Reduced cholesterol efflux was shown in T1D [13, 14], although Vaisar et al. [49] did not find a difference in cholesterol removal comparing subjects with T1D with and without vascular complication. One study showed that improved glycemic control is associated with increased cholesterol efflux in T1D [51]. In this investigation, both HDL_2_ and HDL_3_-mediated cholesterol efflux was similar between T1D and controls and not related to vascular function tests.

### Study strengths and limitations

This is the first demonstration of an individualized and robust proteomic analysis in HDL subfractions with a large number of individuals in a case–control study dealing with T1D subjects. Results can help to better explore HDL proteomics and its predictive role in subclinical atherosclerosis in T1D. Limitations include the fact that the cross-sectional design makes it difficult to draw conclusions about the cause-effect relationship among variables. Considering the heterogeneity of HDL composition and different pathways involved in its generation and catabolism, it is conceivable that many endogenous and exogenous factors may modulate this lipoprotein structure and ultimately function. Hypertension and use of statins were present only in the T1D that could interfere in the case–control study. Moreover, cholesterol efflux to HDL subfractions was determined in a single period of time, which may compromise the inference of HDL functionality over time.

## Conclusions

This study detected differences in HDL proteomics between individuals with T1D and controls that had not been found in previous studies. Nine proteins in HDL_2_, and 22 in HDL_3_ were detected, whose observed abundance differences were not reported in other case–control studies. Vascular tests corroborate previous studies in individuals with T1D that indicated worse markers of subclinical atherosclerosis and higher estimated cardiovascular risk than individuals without diabetes, even with an apparently better serum lipid profile in individuals with T1D. The association of clinical and laboratory variables, HDL proteomics data and results of vascular function tests and HDL functionality made it possible to explore the various atheroprotective functions of HDL. In addition to proteins involved in lipid metabolism, HDL carries acute-phase inflammatory, complement system, antithrombotic, antioxidant, protease inhibitor and transporter proteins, with significant differences between T1D and controls.

## Supplementary Information

**Additional file 1: Table S1.** Proteomics of HDL_2_ e HDL_3_ according to A1c, statin use and cardiovascular autonomic neuropathy in type 1 diabetes.

## Data Availability

The data are available from the corresponding author upon reasonable request. MS proteomics data have been deposited to the Mass Spectrometry Interactive Virtual Environment (MassIVE)—access via ftp://massive.ucsd.edu/MSV000087986/ and Doi: https.doi.org.10.25345/C5MP1C.

## Abbreviations

A1AT: Alpha-1 antitrypsin
A1BG: Alpha-1-B glycoprotein
ABCA-1: ATP binding cassette transporter A-1
ABCG-1: ATP binding cassette transporter G-1
AHSG: Alpha2-Heremans-Schmid glycoprotein or fetuin-A
Alb: Albumin
AMBP: Alpha-1 microglobulin bikunin precursor
APMAP: Adipocyte plasma membrane-associated protein
Apo(a): Apolipoprotein (a)
Apo: Apolipoprotein
ASCVDR: Atherosclerotic cardiovascular disease risk
C3: Complement C3
CAN: Cardiovascular autonomic neuropathy
CETP: Cholesteryl ester transfer protein
CLU: Clusterin, apolipoprotein J or sulfated glycoprotein 2
CST3: Cystatin C
CVD: Cardiovascular disease
d: Density
DCCT/EDIC: Diabetes Control and Complications Trial/Epidemiology of Diabetes Intervention and Complications Study
DM: Diabetes mellitus
FMD: Flow-mediated vasodilation
GC: Vitamin D binding protein or group-specific component
GPLD1: Phosphatidylinositol-glycan-specific phospholipase D
HbA1c: Glycated hemoglobin
HBB: Hemoglobin subunit beta
HDL: High-density lipoprotein
HDL_2_: HDL subfraction 2
HDL_3_: HDL subfraction 3
HDLc: High-density lipoprotein cholesterol
HP: Haptoglobin
HPHPR: Haptoglobin or haptoglobin related protein
IGFALS: Acid-labile subunit
LCAT: Lecithin-cholesterol acyltransferase
LDLc: Low-density lipoprotein cholesterol
Orm1: Alpha-1 glycoprotein 1 or orosomucoid
PCSK9: Proprotein convertase subtilisin/kexin type 9
PCYOX: Prenylcysteine oxidase 1
PLTP: Phospholipid transfer protein
Pon1: Paraoxonase-1
Pon3: Paraoxonase-3
PRM: Parallel reaction monitoring
PWV: Pulse wave velocity
RBP4: Retinol binding protein type 4
RCT: Reverse cholesterol transport
RH: Reactive hyperemia
SAA1: Serum amyloid A type 1
SAA4: Serum amyloid A type 4
T1D: Type 1 diabetes
T2D: Type 2 diabetes
TC: Total cholesterol
TF: Serotransferrin
TG: Triglycerydes
TTR: Transthyretin
VTN: Vitronectin
