# Supplementary material for: Proteomics of high-density lipoprotein subfractions and subclinical atherosclerosis in type 1 diabetes mellitus: a case–control study
Source: Diabetol Metab Syndr. 2023 Mar 11;15:42. doi: 10.1186/s13098-023-01007-y (PMC10007776; doi:10.1186/s13098-023-01007-y)
Supplement: Supplementary file 1 — Additional file 1: Table S1. Proteomics of HDL2 e HDL3 according to A1c, statin use and cardiovascular autonomic neuropathy in type 1 diabetes. [file 13098_2023_1007_MOESM1_ESM.docx]

**Supplemental Table 1. Proteomics of HDL_2_ e HDL_3_ according to A1c, statin use and cardiovascular autonomic neuropathy in type 1 diabetes.**

|  | **A1c** | |  | **Statin use** | | | **Cardiovascular autonomic neuropathy** | | |
| --- | --- | --- | --- | --- | --- | --- | --- | --- | --- |
| **Protein** | **< 8.5%**  **(n = 29)** | **≥ 8.5%**  **(n = 21)** | ***p*** | **(-)**  **(n=30)** | **(+)**  **(n=20)** | ***p*** | **(-)** | **(+)** | ***p*** |
| **A1c (%)** | 5.7 (5.3-7.4) | 9.5 (9.0-11.0) | <0.01 |  |  |  |  |  |  |
| **HDL_2_** |  |  |  |  |  |  |  |  |  |
| **AMBP** |  |  |  |  |  |  | 5.3 (5.2-5.6) | 5.8 (5.4-6.1) | <0.01 |
| **APMAP** | 7.6 (7.3-7.7) | 7.9 (7.7-8.1) | <0.01 |  |  |  |  |  |  |
| **ApoB** | 8.4 (8.0-8.5) | 8.7 (8.4-9.0) | <0.01 |  |  |  | 8.4 (8.2-8.8) | 8.7 (8.5-9.0) | 0.05 |
| **ApoC-I** | 10.0 (9.9-10.0) | 10.1 (10.0-10.1) | 0.04 |  |  |  |  |  |  |
| **C3** | 6.9 (6.7-7.1) | 7.3 (6.9-7.5) | <0.01 | 6.9 (6.8-7.2) | 7.3 (7.0-7.6) | 0.02 |  |  |  |
| **HBB** |  |  |  | 6.1 (5.4-6.6) | 6.7 (6.4-6.9) | <0.01 |  |  |  |
| **IGFALS** | 5.7 (5.2-6.6) | 5.6 (4.7-6.0) | 0.05 |  |  |  |  |  |  |
| **Lp(a)** |  |  |  | 8.3 (8.0-8.5) | 8.7 (8.5-8.9) | <0.01 | 8.5 (8.0-8.7) | 8.7 (8.4-8.9) | 0.03 |
| **Orm1** |  |  |  |  |  |  | 5.6 (5.0-6.0) | 5.9 (5.5-6.4) | 0.03 |
| **SAA4** | 9.2 (9.1-9.3) | 9.3 (9.1-9.4) | 0.04 |  |  |  |  |  |  |
| **TTR** |  |  |  |  |  |  | 7.7 (7.3-7.8) | 7.8 (7.5-8.0) | 0.04 |
| **HDL_3_** |  |  |  |  |  |  |  |  |  |
| **A1AT** | 10.1 (9.9-10.2) | 10.0 (9.6-10.1) | <0.01 |  |  |  |  |  |  |
| **A1BG** | 7.8 (7.6-8.0) | 7.6 (7.4-7.9) | 0.04 |  |  |  |  |  |  |
| **Alb** | 10.3 (10.1-10.6) | 10.0 (9.7-10.3) | <0.01 |  |  |  |  |  |  |
| **APMAP** | 7.2 (6.5-7.8) | 7.8 (7.5-8.0) | <0.01 |  |  |  |  |  |  |
| **ApoA-I** | 11.0 (10.6-11.1) | 11.1 (11.0-11.1) | <0.01 |  |  |  |  |  |  |
| **ApoA-II** | 9.8 (9.5-10.1) | 10.1 (10.0-10.2) | <0.01 |  |  |  |  |  |  |
| **ApoA-V** | 7.6 (7.4-7.8) | 7.7 (7.6-7.9) | 0.02 |  |  |  |  |  |  |
| **ApoC-I** | 9.6 (9.3-9.8) | 9.6 (9.6-9.8) | 0.04 |  |  |  |  |  |  |
| **ApoC-II** | 9.2 (8.9-9.4) | 9.4 (9.2-9.5) | 0.02 |  |  |  |  |  |  |
| **ApoC-III** | 9.5 (9.2-9.7) | 9.7 (9.5-9.9) | <0.01 |  |  |  |  |  |  |
| **ApoD** | 9.6 (9.4-9.9) | 9.8 (9.7-9.9) | <0.01 |  |  |  |  |  |  |
| **ApoF** | 9.3 (9.0-9.7) | 9.1 (8.8-9.2) | 0.02 |  |  |  |  |  |  |
| **ApoM** | 9.3 (9.2-9.5) | 9.5 (9.4-9.5) | <0.01 | 9.5 (9.4-9.6) | 9.4 (9.4-9.5) | 0.02 |  |  |  |
| **CLU** |  |  |  |  |  |  | 8.7 (8.5-8.8) | 8.5 (8.4-8.6) | 0.02 |
| **GC** | 8.2 (7.9-8.4) | 7.7 (7.3-8.1) | <0.01 |  |  |  |  |  |  |
| **HBB** | 7.6 (7.4-8.0) | 7.3 (7.1-7.6) | <0.01 |  |  |  | 7.4 (7.3-7.5) | 7.3 (7.1-7.5) | 0.05 |
| **HPHPR** | 7.8 (7.6-8.0) | 8.0 (7.9-8.2) | 0.02 |  |  |  |  |  |  |
| **IGFALS** |  |  |  | 7.1 (6.8-7.3) | 6.5 (6.2-7.1) | 0.04 |  |  |  |
| **PCSK9** | 7.3 (7.0-7.6) | 7.5 (7.3-7.6) | 0.02 |  |  |  |  |  |  |
| **PON3** |  |  |  | 7.6 (7.4-7.8) | 7.5 (7.3-7.6) | 0.02 | 7.6 (7.5-7.8) | 7.4 (7.3-7.5) | <0.01 |
| **RBP4** | 8.6 (8.5-8.7) | 8.6 (8.3-8.6) | 0.04 |  |  |  |  |  |  |
| **SAA4** | 9.0 (8.8-9.2) | 9.2 (9.0-9.4) | <0.01 |  |  |  |  |  |  |
| **TTR** | 9.1 (9.0-9.1) | 8.9 (8.7-9.0) | <0.01 |  |  |  |  |  |  |

Median and 25th and 75th percentiles of protein abundance, in log10, Mann-Whitney test.A1AT: alpha1 antitrypsin; A1BG: alpha-1-B glycoprotein; Alb: albumin; AMBP: alpha 1 microglobulin bikunin precursor; APMAP: adipocyte plasma membrane-associated protein; Apo: apolipoprotein; CAN: cardiovascular autonomic neuropathy; C3: complement C3; CLU: clusterin or apolipoprotein J; GC: vitamin D binding protein or group-specific component; HBB: hemoglobin subunit beta; HPHPR: haptogolobin related protein or haptoglobin; IGFALS: acid-labile subunit; Lp(a): apolipoproteína(a); Orm1: alpha -1 glycoprotein 1 or orosomucoid; PCSK9: proprotein convertase subtilisin/kexin type 9; PON3: paraoxonase-3; RBP4: retinol binding protein type 4; SAA4: serum amyloid A type 4; TTR: transthyretin.
